# mSphere Legacy: Fritz about Fritz

**DOI:** 10.1128/msphere.00361-26

**Published:** 2026-07-15

**Authors:** Friedrich Götz

**Affiliations:** 1 Microbial Genetics, Interfaculty Institute of Microbiology and Infection Medicine Tübingen (IMIT), University Tübingen 9188, Tübingen, Germany; University of Nebraska Medical Center College of Medicine, Omaha, Nebraska, USA

**Keywords:** legacy commentary, Friedrich Götz, Fritz

## Abstract

Looking back over the past 50 years, staphylococcal research has evolved from a relatively narrow niche into a broad, interdisciplinary field encompassing genetics, genomics, bioinformatics, host–bacterium interactions, immunity, microbial ecology, the microbiota, and commensalism. My career progressed in parallel with these research developments. Much of what contributed to my success was the ability to recognize emerging questions early on, to combine classical microbiology with new technologies as they emerged, and to collaborate with talented students and colleagues across disciplines. Despite the diversity of the topics I have explored, my work has consistently been driven by a central question: what do staphylococci do, and how do they manage to persist so tenaciously? A brief overview of my research interests may illustrate this trajectory.

## COMMENTARY

## FIRST STEPS IN SCIENCE/RESEARCH

I began my research career in the late 1970s with my doctoral thesis at LMU Munich under Karl Heinz Schleifer in the Department of Microbiology. At the time, the department was a hotspot for bacterial classification, cell wall structure analysis, staphylococcal physiology, and metabolism. I became particularly interested in the latter areas and studied fructose-1,6-bisphosphate (FBP)-activated L-lactate dehydrogenase in *Staphylococcus epidermidis* and a highly stable class I FBP aldolase in *Staphylococcus aureus* (1). I also investigated oxygen consumption mediated by pyruvate oxidase in *Lactobacillus plantarum*, demonstrating that this bacterium does not require an enzymatic superoxide dismutase because it can use Mn-phosphate/pyrophosphate for superoxide scavenging—an example of the diverse mechanisms that bacteria employ to cope with oxidative stress (2–4). My scientific environment was shaped by the work of Karl-Heinz Schleifer (Technical University of Munich) and Wesley E. Kloos (North Carolina State University) who made foundational contributions to the identification and classification of *Staphylococcus* species, particularly during the 1970s, when bacterial systematics was undergoing major modernization. The Kloos–Schleifer classification frameworks remained standard references for the identification of staphylococci well into the molecular era (5, 6).

Because of the growing antibiotic resistance crisis, the first “International Symposium on Staphylococci and Staphylococcal Infections” (ISSSI) was held in Paris in 1963. Later, under the leadership of Janusz Jeljaszewicz, the ISSSI was institutionalized and continued in Warsaw, giving the conference a unique role as a Cold War “scientific bridge.” Warsaw hosted the second ISSSI in 1971 and subsequently every meeting through the sixth ISSSI in 1989. For us young scientists, these meetings revealed that there truly was an international staphylococcal community—one that was focused not only on clinical aspects, but also highly receptive to fundamental research. At the same time, there was the added curiosity of catching a glimpse behind the Iron Curtain.


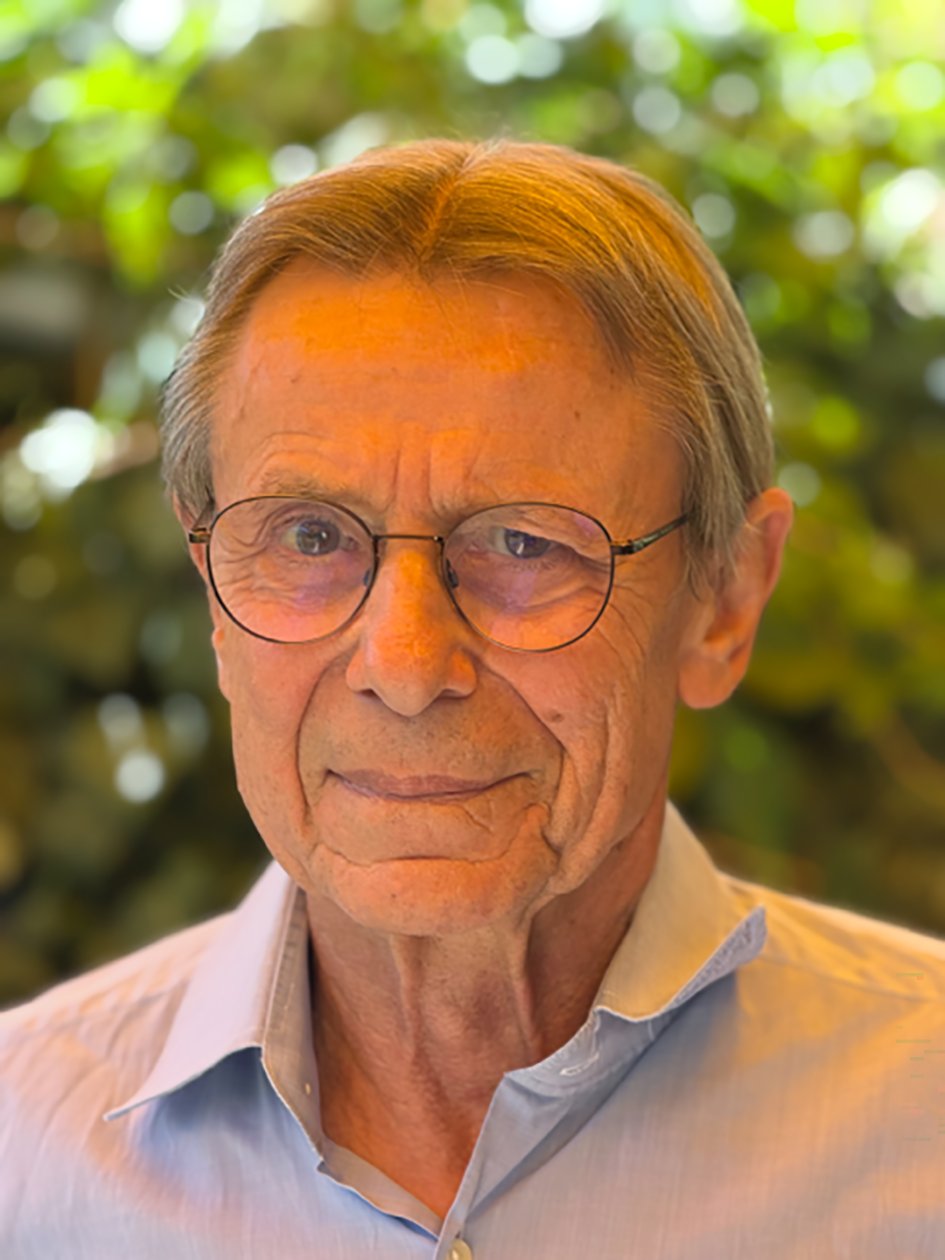


As the ISSSI was primarily clinically oriented, the Gordon Research Conference (GRC) on Staphylococcal Diseases was established in 1989 with a biennial meeting cycle. The inaugural GRC on Staphylococcal Diseases was chaired by Richard Proctor in 1989 at Salve Regina University. The meeting was then held under the title “Staphylococcal Pathogenesis.” In contrast to the ISSSI, this GRC conference placed a stronger emphasis on fundamental and mechanistic aspects of staphylococcal research.

## THE MOLECULAR BIOLOGY OF STAPHYLOCOCCI WAS TAKING SHAPE

Sir Mark Richmond (Bristol, UK) investigated the role of β-lactamase-encoding plasmids in *S. aureus* and helped shape our understanding of how antibiotic resistance spreads and evolves (7). He also worked with *S. aureus* 8325 (NCTC 8325 or RN1), a human bloodstream isolate originally used in the UK for phage typing that later became one of the earliest model strains for studying staphylococcal molecular genetics worldwide (8). Edward Rosenblum (University of Texas) focused on classical genetics, phage biology, and transduction (8, 9). Peter A. Pattee (Iowa State University) helped transform *S. aureus* research from a primarily medical bacteriology discipline into a genetically defined model system for the study of virulence. Richard Marples (UK) was among the first to systematically study staphylococci as commensals rather than solely as pathogens. Wesley E. Kloos (North Carolina State University) mapped the distribution of staphylococcal and micrococcal species on human skin (10). Richard Novick (New York University School of Medicine) was one of the foundational figures in the modern molecular genetics of staphylococcal infections. He developed important cloning vectors, transduction systems, and plasmid-based tools that enabled modern staphylococcal genetics, and he also elucidated key aspects of plasmid replication mechanisms (11). Many later researchers built directly upon these methods. Probably his most influential contribution was the analysis of the accessory gene regulator (*agr*) system, the major quorum-sensing regulator controlled by autoinducing peptides (AIPs), which governs the switch between colonization and invasive virulence through the regulation of toxins, adhesins, and secreted factors (12). Martin Lindberg (Uppsala, Sweden) was another pioneer who helped establish modern molecular genetics in staphylococcal research during the 1960s–1980s. He developed genetic transformation systems, advanced the understanding of plasmid and phage biology, and contributed to the characterization of the gene encoding staphylococcal protein A (*spa*), one of the most important immune-evasion proteins and later a widely used biotechnology tool (13, 14). This endeavor was part of the general rise of recombinant DNA technology, which I found both fascinating and immensely powerful as a means of solving the many open questions in the field of bacterial physiology.

## LEARNING MOLECULAR CLONING AT BMC, UPPSALA

To learn DNA cloning in staphylococci, I went to work as a postdoctoral researcher in the group of Martin Lindberg and Lennart Philipson at the Uppsala Biomedical Center (BMC), Sweden, from 1979 to 1981. It was a pioneering period, and many of the essential tools had yet to be developed: methods for DNA isolation, restriction digestion, ligation, transformation, and the use of antibiotic resistance genes as selectable markers. Restriction enzymes were not yet commercially available and had to be isolated directly from bacteria. Here, my previous experience in enzyme isolation/purification proved highly useful. Richard Novick of the Public Health Research Institute of the City of New York (PHRI) was the acknowledged master of plasmid biology. He was one of my scientific heroes and generously provided us with his plasmids as cloning vectors.

In 1980, during my stay at the BMC, Staffan Arvidson of the Karolinska Institute in Stockholm presented an astonishing talk about a pleiotropic *S. aureus* mutant (15). It was later shown that the mutation affected the *agr* regulon, a global regulatory system controlled by a quorum-sensing mechanism, as subsequently unraveled in Richard Novick’s group. The transformation from descriptive to molecular, genetic, and mechanistic microbiology was equally evident in staphylococcal research. The 1980s and 1990s witnessed the PCR revolution and continuous advances in DNA sequencing, which became increasingly rapid and affordable, driving enormous growth in cloning, recombinant DNA technology, genomics, microbial biotechnology, and diagnostics.

When I returned from Uppsala to the Technical University of Munich (TUM) in 1981, joining the group of Karl Heinz Schleifer, I set out to establish a cloning system in the food-grade species *Staphylococcus carnosus* to study pathogenic traits of *S. aureus* in a non-pathogenic host. At first, the project was unsuccessful. All the tools developed for *S. aureus* failed in *S. carnosus*. Just as I was about to give up, I finally made a breakthrough. One of the first genes cloned in *S. carnosus* was the phospholipase gene of *Staphylococcus hyicus*. This proved to be a key discovery, showing that staphylococcal lipases are synthesized as pre-pro-enzymes and that the pro-region functions as an intramolecular chaperone, which can be used as a (“chassis”) for the expression and export of heterologous proteins in *S. carnosus*. What we learned from these early synthetic biology approaches was that a cloning system for one bacterial species could not easily be transferred to another. Unknown DNA- and RNA-based restriction systems slowed progress while at the same time opening new scientific questions.

Besides the colleagues mentioned in the text, there are quite a number of current and former cooperation and discussion partners:

- Arnold S. Bayer*—S. aureus* bacteremia and infective endocarditis
- Kenneth W. Bayles—physiology of *S. aureus*, programmed cell death
- Nadia Berkova*—S. aureus* reprograms host cell cycles
- Ambrose L. Cheung—discovery of staph accessory regulator (sar) system
- Jiri Doskar—isolation and characterization of *S. aureus* bacteriophages
- Paul D. Fey—mechanisms of staphylococcal biofilm formation
- Simon J. Foster—peptidoglycan architecture and cell wall structure
- Timothy J. Foster—staphylococcal adhesins (MSCRAMMs)
- Anneliese Gründling—staphylococcal cyclic-Di-AMP
- Keiichi Hiramatsu—characterization of the SCCmec element
- Regine Landmann—host response in a mouse tissue cage infection model
- Jodi Lindsay—evolution of MRSA
- Shih-Tung Liu—biosynthesis of fengycin
- Tao Jin—pathogenesis of *S. aureus* in septic arthritis and sepsis
- James O'Gara—biofilm formation and persistence
- Knut Ohlsen—bacterial virulence in murine models
- Francoise Perdreau-Remington—community associated USA300
- Gerald Pier—vaccine development targeting PIA/PNAG
- Mariana G. Pinho—coordination of cytokinesis in spherical cocci
- Steven Projan—anti-infective drug discovery
- Richard A. Proctor—discovery of small-colony variants (SCVs)
- Di Qu—biofilm-associated proteins in *S. epidermidis*
- Olaf Schneewind—discovery of sortase and surface protein anchoring
- Dominique Missiakas—sortase biology and virulence proteins
- Eric P. Skaar—bacterial struggle for iron and zinc in the host environment
- Motoyuki Sugai—cell surface architecture of *S. aureus*
- Alex Tomasz*—mecA* and peptidoglycan structure
- François Vandenesch—CA-MRSA lineages and PVL genes
- Hubertus M. Verheij—activity and enantioselectivity of lipases
- Christiane Wolz—regulatory networks and stress adaptation control
- Michael R. Yeaman—innate immune defense mechanisms against *S. aureus*
- Wenqi Yu—surface localization of *S. aureus* virulence proteins
- Wilma Ziebuhr—mutations in staphylococcal biofilm formation

## APPOINTMENT TO THE CHAIR OF “MICROBIAL GENETICS” IN TÜBINGEN

When I was appointed to the Chair of Microbial Genetics at the University of Tübingen in 1987, the debate over the risks of recombinant DNA technology was still intense. As someone directly involved in the field and as a newly appointed professor, I had to participate in numerous hearings and panel discussions, where I tried as best I could to bridge entrenched positions. The legendary Asilomar Conference on Recombinant DNA (1975), at which leading scientists developed safety guidelines for genetically modified organisms (GMOs), had taken place a decade earlier. Nevertheless, many fears remained, and we had to address them seriously. At times, it even seemed possible to me that I might not be able to continue my work in Tübingen. Tensions eased noticeably when the International Center for Ethics in the Sciences and Humanities (IZEW) was founded there in 1990.

## I WAS PARTICULARLY FASCINATED BY PHYSIOLOGY AND BIOSYNTHETIC PATHWAYS

### Lantibiotics: epidermin and gallidermin biosynthesis

Lantibiotics are ribosomally produced peptide antibiotics that contain lanthionine rings formed through post-translational modification. When I arrived in Tübingen, structural analyses of epidermin isolated from *S. epidermidis* by the group of Hans Zähner and of Pep5 isolated by Hans Georg Sahl (University of Bonn) were underway in the group of Günther Jung in organic chemistry. A landmark 1988 study identified the prepeptide gene sequence of epidermin, showing that it is produced ribosomally as a precursor peptide, and then enzymatically modified into the active antibiotic (16). This was a major conceptual advance because it revealed how these unusual molecules are biosynthesized. Gallidermin subsequently became one of the best-studied lantibiotics because of its strong anti-staphylococcal activity (17). The term “lantibiotics” (lanthionine-containing peptide antibiotics) soon became standard in microbiology.

Among the first master and PhD students in Tübingen were Andreas Peschel, Michael Otto, and Thomas Kupke. They worked on elucidating the biosynthesis of epidermin through the *epi* genes, later renamed *lan* genes for lantibiotics. In the following years, together with other students, they unraveled the functions and mechanisms of the ten *lan* genes involved in post-translational modification, transport, immunity, processing, and regulation (18–22). Today, lantibiotics are recognized as part of the growing class of ribosomally synthesized and post-translationally modified peptides (RiPPs). Andreas established his own group and eventually became my successor. His team discovered key staphylococcal resistance mechanisms against antimicrobial peptides, including MprF and the Dlt operon. They also clarified how neutrophils, antimicrobial peptides, and skin immune defenses interact with *S. aureus*, thereby establishing the concept that successful staphylococcal pathogens are highly adapted to withstand human innate immunity rather than merely relying on toxin production. Michael discovered and characterized the phenol-soluble modulins (PSMs) as central toxins and signaling peptides in *S. aureus*. He further demonstrated the direct link between Agr activity, PSM expression, and invasive disease potential. He pursued his career at the National Institutes of Health in Bethesda.

### Molecular basis of staphylococcal biofilm

In the 1980s and 1990s, Gordon D. Christensen (University of Tennessee College of Medicine, USA) linked slime production to the pathogenicity of *S. epidermidis*, while Georg Peters (Institute of Medical Microbiology, University Hospital Münster, Germany) made major mechanistic contributions to biofilm components, including slime and surface proteins, such as accumulation-associated protein (AAP). Their work paved the way for the discovery of slime as a linear β-1,6-linked glucosaminoglycan (PIA, also called PNAG) by Mack et al. in 1996 (23). In the same year, Christine Heilmann in our lab identified and characterized the *ica* operon encoding the biosynthesis genes for PIA/PNAG (24). This is regarded as one of the most important papers from Fritz’s group. Later, *ica* orthologous genes were identified in many pathogens, including *Staphylococcus aureus*, *Escherichia coli*, *Actinobacillus pleuropneumoniae*, *Acinetobacter baumannii*, or *Enterococcus faecalis*.

The central problem of biofilm-associated implant infections is that bacteria are surface-attached, protected by matrix material, metabolically heterogeneous, partially dormant, and shielded from host immunity. Embedded in this matrix, the supposedly benign commensal *S. epidermidis* turns into a recognized biofilm pathogen (25). The study of biofilms occupied us for many years. Our work contributed to defining the modern biological model of staphylococcal biofilms: how they form, what their matrix consists of, how they are regulated, why they resist treatment, and why they are clinically important in chronic implant and device infections. Our findings also paved the way for treatment strategies against staphylococcal biofilm infections.

### Nitrate respiration and staphyloxanthin pathways

While nitrate respiration had been well studied in gram-negative bacteria, little was known in staphylococci when we began to investigate this topic using *S. carnosus* as a model organism. We showed that the reduction of nitrate to ammonia occurs in two stages, and we also identified a novel oxygen-sensitive regulatory system, NreABC, controlling nitrate/nitrite respiration genes (26, 27). NreB functions as an oxygen sensor, allowing staphylococci to switch to nitrate respiration when oxygen becomes scarce, such as in partly anoxic infection sites or during co-infection with other bacteria (28). Staphyloxanthin is a hallmark of *S. aureus*. In my lab, we have elucidated its biosynthetic pathway (*crtOPQMN*), its NMR structure, and its role in coping with oxidative stress (29, 30).

### Lipoprotein immune modulation and host cell invasion

Over many years, we worked to better understand the role of lipoproteins in staphylococci, especially in *S. aureus*. We showed that these molecules are not merely membrane-anchored proteins but major factors in nutrient uptake, immune activation, persistence, and immune evasion (31). One landmark finding came from studies of *lgt* mutants lacking lipoprotein diacylglyceryl transferase, the enzyme responsible for lipidating prelipoproteins. We demonstrated that when *S. aureus* cannot properly lipidate its lipoproteins, immune stimulation is greatly reduced; host inflammatory responses are blunted; and bacterial fitness is impaired (32, 33). This established that the lipid moiety of lipoproteins is crucial for immune recognition and essential for bacterial survival through metal ion acquisition and the chaperone YidC. Minh-Thu Nguyen showed that di- and tri-acylated lipopeptides trigger distinct immune responses via either TLR2/TLR6 or TLR2/TLR1 (34, 35).

One group of lipoproteins attracted our special attention: the Lpl lipoproteins. These form a distinct class with multiple paralogous *lpl* genes clustered on specific genomic islands, especially in epidemic *S. aureus* strains. We discovered that they have an important previously unknown role in virulence (36, 37). Functional analysis of the recombinant model protein Lpl1 revealed that it delays the G2/M transition in host cells, and Paula Tribelli further showed that Lpl1 binds to the human Hsp90 isoforms Hsp90α and Hsp90β, thereby promoting invasion of epithelial cells, an effect enhanced at 39°C (fever temperature) (38, 39). The interaction of Lpl surface proteins with Hsp90 chaperones, thus, represents a new host–pathogen interaction pathway in *S. aureus*.

### MpsABC bicarbonate transporter is essential in *S. aureus*

The story of MpsABC began in 2015 with earlier work in our laboratory by Sonja Maier, who investigated whether *S. aureus* possesses a type I electrogenic NADH:quinone oxidoreductase (Ndh1), a cation-translocating proton pump. Such an Ndh1 is absent in staphylococci. Instead, we identified a hypothetical protein in *S. aureus* with sequence similarity to the *Escherichia coli*-specific NuoL protein, one of the core components of the proton-pumping machinery (40). The corresponding *nuoL*-like gene in *S. aureus* was named *mpsA* (membrane potential-generating system) because deletion mutants were severely impaired in membrane potential generation and exhibited a small-colony variant-like phenotype. *mpsA* is the first gene of an operon comprising *mpsA*, *mpsB*, and *mpsC*. However, *mpsC* is separated from *mpsB* and dispensable for function (41). Subsequently, Sook-Ha Fan and Elisa Liberini showed that MpsAB is essential for growth at normal atmospheric CO_2_ levels and functions as a bicarbonate transporter/carbon-concentrating system required for *S. aureus* growth and virulence (42–44). MpsAB, thus, emerged from a poorly understood operon into a recognized possible therapeutic target in staphylococcal physiology and pathogenesis.

### Neurotransmitter-producing microbiota and host interaction

Our contribution to trace amine (TA)-producing microbiota lies at the intersection of staphylococcal and commensal metabolism, amino acid decarboxylation, and host–microbe signaling. Core discoveries by Arif Luqman and Samane Rahmdel showed that skin-associated staphylococci and other commensals are not metabolically inert but actively produce neuroactive aromatic monoamines (NAAMs), including tyramine, phenethylamine, tryptamine, and dopamine, in some strains (45–47). These compounds are generated particularly through aromatic amino acid decarboxylation pathways (48, 49). Our work supports the idea that commensal-derived trace amines may interact with trace amine-associated receptors (TAARs) (especially TAAR1-like pathways), influence keratinocyte and immune-cell signaling, modulate local inflammatory tone in the skin, and link microbial metabolism to host immunity. Trace amines and dopamine may also contribute to wound healing by enhancing cell migration and re-epithelialization of keratinocytes (50). The interaction of commensal metabolites with host neuro- and G protein-coupled receptors will continue to occupy our attention for some time.

## MY ADVICE TO THE NEXT GENERATION OF MICROBIOLOGISTS

### Grant applications: a never-ending struggle

Securing external funding is an unavoidable part of every scientist’s professional life. I am deeply grateful for the continuous support of my research, particularly from the German Research Foundation (DFG). Most of my grant applications have been successful. Perhaps, this was also because my funding requests were realistic and never excessive. My motto has always been “A bird in the hand is worth two in the bush.” I also experienced that some research proposals were rejected by reviewers. In most cases, these involved interdisciplinary topics, which are, in principle, politically encouraged. However, one easily runs into the dilemma of not being regarded as an expert in the other field. Caution is therefore required in such cases. Some rejected proposals were subsequently pursued with alternative funding and ultimately became highly successful research projects.

### How to choose scientific questions

Choosing a good scientific question is one of the most important and difficult parts of research. My scientific questions were often inspired by gaps or inconsistencies in prevailing models: observations that did not quite fit, clinical problems lacking mechanistic explanations, or unexpected phenotypes hinting at hidden regulatory layers. Curiosity, combined with a willingness to pursue such clues, shaped the direction of my work. I was fortunate to work with many exceptionally talented and motivated students, far too many to mention here. I tried to give them as much freedom as possible to develop their own ideas. Asking the right questions and developing or adapting new methods helped us move forward.

### “Open science” greatly accelerates scientific progress

The staphylococcal research community has greatly benefited from the principles of “open science,” which promote the unrestricted sharing of scientific knowledge, data, methodologies, software, and educational resources within and beyond the scientific community. Several notable examples illustrate the impact of this approach.

Early efforts to sequence the *S. aureus* genome were initially carried out in more restricted, proprietary settings, and the resulting data were not always made broadly available to the scientific community. This lack of open access prompted the NIH to support publicly funded genome sequencing initiatives in the late 1990s, ensuring that *S. aureus* genomic data would be generated and shared freely to enable wider use in infectious disease research. However, the public release of the first *S. aureus* N315 and Mu50 genome sequences has been published by Kuroda et al. in 2001 and represents an excellent example of a freely available resource that catalyzed research worldwide (51).

We have learned the importance of model strains for answering fundamental biological questions. *S. aureus* USA300 has emerged as another global model organism. It possesses many major virulence factors and is multidrug resistant. Based on this strain, Kenneth W. Bayles and Paul Fey helped create the Nebraska Transposon Mutant Library (NTML) (52). This library contains mutations in nearly all non-essential genes and has become a standard tool used worldwide to study staphylococcal gene function, virulence, metabolism, and antibiotic resistance.

At the University of Greifswald, the groups of Michael Hecker, Uwe Völker, and Ulrike Mäder established large-scale proteomics approaches for *S. aureus*. At a time when genomics dominated microbiology, this group focused on identifying and quantifying the proteins expressed under different physiological conditions. They also initiated AureoWiki, a curated knowledge base for *S. aureus* that is freely available to researchers worldwide (*53*).

These efforts marked a milestone in two ways: first, by providing a powerful tool that saved researchers enormous amounts of time; second, by demonstrating that science can also be organized in the common interest rather than purely individual competition.

Bacteriology has changed profoundly over these decades. Technologies, such as microbiome analysis, metagenomics, and bioinformatic tools, like the structure-prediction platform AlphaFold, have greatly accelerated progress. The shift from phenotypic to molecular approaches, and later to genome-wide system-level analyses, continues to reshape our understanding of *S. aureus* and other pathogens. With each technological leap, new concepts emerge; new questions become answerable; and older assumptions must be re-examined. Given enough time, I would love to investigate how bacteria develop forms of collective memory.

A scientist is seldom at rest, for they know that today’s radiant insight may soon fade in the light of deeper understanding.
